# Ion mobility mass spectrometry for the study of mycobacterial mycolic acids

**DOI:** 10.1038/s41598-023-37641-9

**Published:** 2023-06-27

**Authors:** Yi Liu, Nadhira Kaffah, Sufyan Pandor, Mark J. Sartain, Gerald Larrouy-Maumus

**Affiliations:** 1 Centre for Bacterial Resistance Biology, Department of Life Sciences, Faculty of Natural Sciences, Imperial College London, London, SW7 2AZ UK; 2 Agilent Technologies, Inc., Cheadle, UK; 3 Agilent Technologies, Inc., Santa Clara, CA 95051 USA

**Keywords:** Mass spectrometry, Lipids

## Abstract

Lipids are highly structurally diverse molecules involved in a wide variety of biological processes. The involvement of lipids is even more pronounced in mycobacteria, including the human pathogen *Mycobacterium tuberculosis*, which produces a highly complex and diverse set of lipids in the cell envelope. These lipids include mycolic acids, which are among the longest fatty acids in nature and can contain up to 90 carbon atoms. Mycolic acids are ubiquitously found in mycobacteria and are alpha branched and beta hydroxylated lipids. Discrete modifications, such as alpha, alpha’, epoxy, methoxy, keto, and carboxy, characterize mycolic acids at the species level. Here, we used high precision ion mobility-mass spectrometry to build a database including 206 mass-resolved collision cross sections (CCSs) of mycolic acids originating from the strict human pathogen *M. tuberculosis*, the opportunistic strains *M. abscessus*, *M. marinum* and *M. avium,* and the nonpathogenic strain *M. smegmatis*. Primary differences between the mycolic acid profiles could be observed between mycobacterial species. Acyl tail length and modifications were the primary structural descriptors determining CCS magnitude. As a resource for researchers, this work provides a detailed catalogue of the mass-resolved collision cross sections for mycolic acids along with a workflow to generate and analyse the dataset generated.

## Introduction

Mycolic acids are long-chain fatty acids and comprise the inner leaflet of mycobacterial outer membranes in cell envelopes^1–5^. They occur mostly in association with sugar moieties and are either covalently linked to arabinogalactan or esterifying trehalose and glycerol; however, they may also exist freely^3,4,6,7^. Mycolic acids are important not only for mycobacterial cell envelope architecture but also for virulence and antibiotic resistance^2,8,9^. Mycolic acids determine cell envelope fluidity and create a hydrophobic layer in association with sugar moieties and lipids; this layer acts as a barrier for drug penetration, thereby conferring intrinsic resistance to antibiotics^10^. The synthesis of mycolic acids is targeted by the first-line anti-TB drug isoniazid through inhibiting the activities of InhA and KasA. Moreover, mycolic acids manipulate the host immune response^7^ and induce foamy macrophage formation characteristic of *Mycobacterium* spp.^11^.

The role of mycolic acids in virulence is determined by their structure. All mycolic acids share a main acyl chain, called the meromycolic chain, and an alpha branch^4,5^. Mycolic acids are divided into different classes according to the functional groups attached to the main meromycolic chain^4–6,9,12^. However, mycolic acid classes are species-specific and not present in all *Mycobacterium* species^8,9,13^. For example, the nonpathogenic mycobacterium *Mycobacterium smegmatis* mc^2^155 does not possess keto and methoxy groups, as found in the pathogenic mycobacterium strain *Mycobacterium tuberculosis* H37Rv^9,14,15^. Additionally, although epoxy mycolic acids are found in *M. smegmatis* mc^2^155, they are not present in *M. bovis* BCG, the vaccine strain^9,14,16^.

Recent studies have shown that mycolic acid chain length, functional groups and structures can influence the fluidity of the mycobacterial cell envelope, which in turn affects drug susceptibility^10^. Furthermore, the chemical functions on the meromycolic chain determine virulence^1,9^. For example, only mycobacteria with oxygenated mycolic acids can induce formation of foamy macrophages, which facilitate mycobacterial persistence within host macrophages^11^. Specifically, keto-mycolic acids are able to interact with the host lipid sensor TR4 and induce this foamy phenotype^17^. Methoxy and keto mycolic acids are also more antigenic than alpha mycolic acids^18^.

It is important to characterize the mycolic acid profiles of different mycobacterial species at a fine structural level to clarify their roles during infection and antimicrobial resistance between different species. Mycolic acids of the same classification may differ in the position and geometry of their chemical functions, adding to their structural complexity. Numerous studies have reported on the identification of mycolic acid classes in different mycobacterial species^12,15,19^ and investigated the roles of mycolic acid classes in virulence^11,17,18^. These approaches involve mass spectrometry techniques, such as matrix-assisted laser desorption ionization^15^ or electrospray ionization^20–22^ mass spectrometry.

However, none of these studies have investigated the use of ion mobility coupled to mass spectrometry to gain deeper insight into mycolic acids. Ion mobility-mass spectrometry (IM-MS) is a technique used to achieve molecular ion separation based on ionic mass, charge, and conformations in the gas phase^23–25^. In contrast to conventional mass spectrometry, the additional information on conformation, which is measured in terms of collisional cross section (CCS), improves the accuracy of identification of molecules with similar structures. Indeed, through ion mobility, the rotationally averaged CCS of an ion can be measured. CCS is an important distinguishing characteristic of an ion in the gas phase. It is related to its chemical structure and three-dimensional conformation.

This method has been previously used to identify lipid isomers, which are lipids that exhibit the same mass but different structures (hence, different CCS values), and classify them according to lipid head group, chain length, and degree of unsaturation^26–29^.

In this study, we focus on the relationship between lipid structure and gas-phase conformation via IM-MS analysis to generate an atlas of mycolic acid methyl ester (MAME) isomers in relevant mycobacterial species.

## Results and discussion

### Data processing workflow

A subset database from online sources (https://www.lipidmaps.org/) and the literature was created in comma-separated values (CSV) format^15,30–32^. Analysis of fragmentation data from the pooled quality control samples performed in Mass Hunter Qualitative Analysis (Agilent Technologies) was used to extract compounds with corresponding spectral information. Data were placed into Sirius 4 (Lehrstuhl Bioinformatik Jena) for molecular formula assignment. Fragment formulas were added to the database^33,34^. Finally, all compounds in the database were annotated with CCS using Mass Profiler (Agilent Technologies).

**Figure 1:**
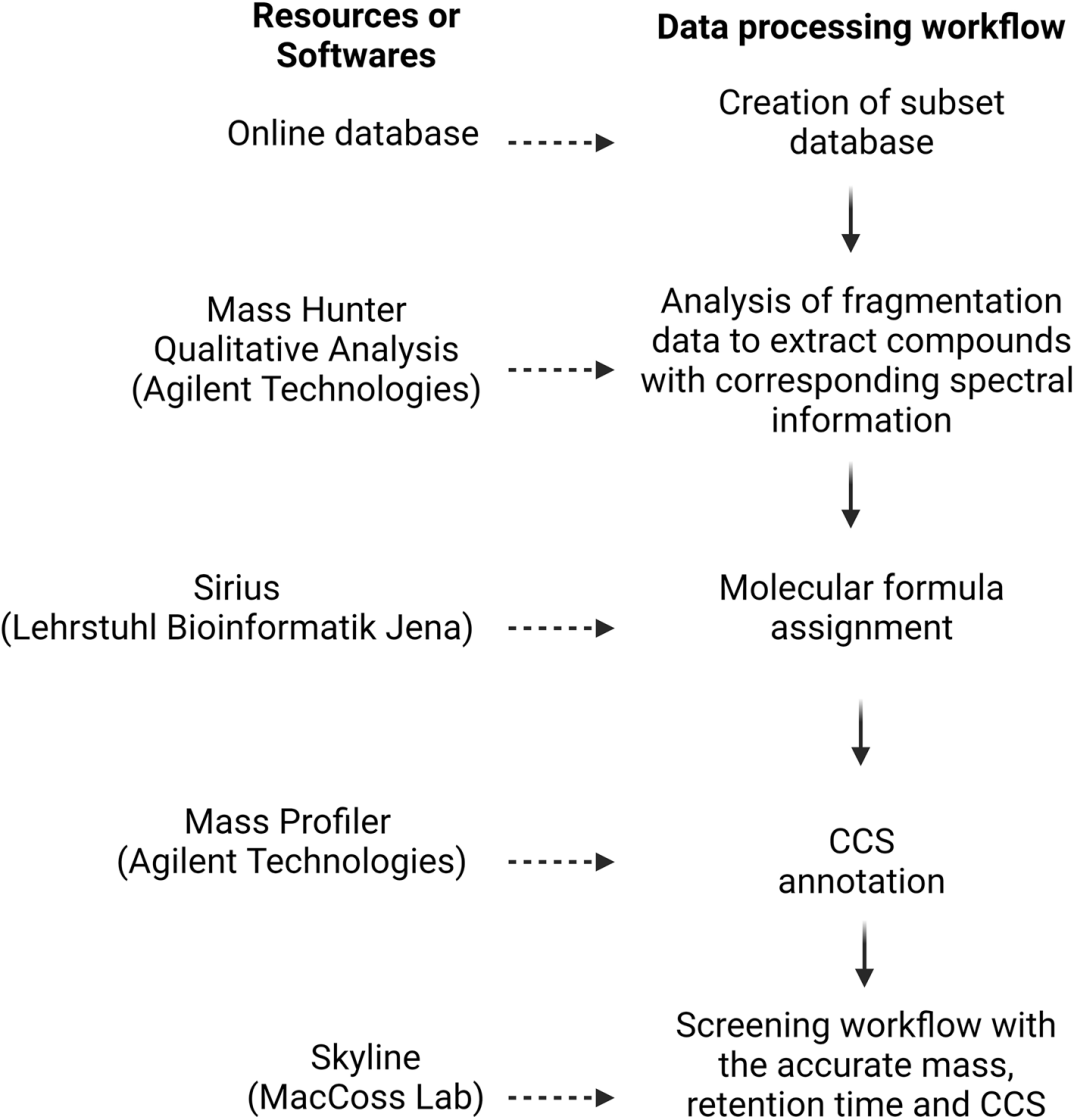
Data process workflow used to extract and annotate the mycolic acid methyl esters (MAMEs) with formula and CCS.

A screening workflow was set up in Skyline (MacCoss Lab), in which all mycolic acids were extracted from IM-QToF^35^. All Ions MS/MS data were collected with accurate mass, retention time and CCS (Fig. 1). Data were filtered to exclude matches with an isotope dot product score lower than 0.8 and a mass error of > 10 ppm.

### Mycolic acid distribution and ion forms

MAMEs were identified and assigned primarily based on exact mass measurements and MS/MS profiles. The CCS measurements and corresponding mobility-mass correlations were utilized to provide additional confidence in the lipid assignment in conjunction with the mass measurement accuracy (± 10 ppm observed in this work).

**Figure 2:**
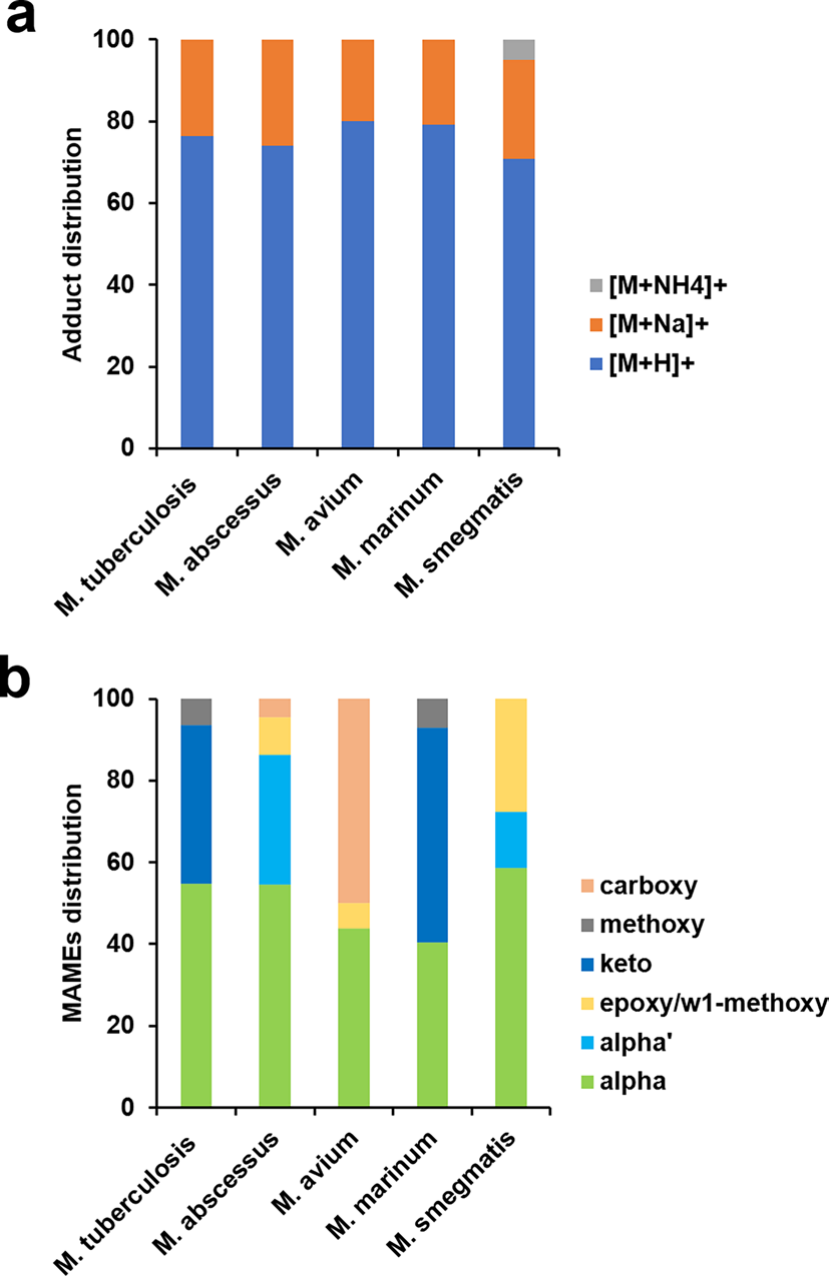
Mycolic acid methyl ester population observations. (**a**) The distribution of adducts observed resulting in the species of quasimolecular ion detected. (**b**) Mycolic acid methyl ester modification distributions across 5 mycobacterial species, *M. tuberculosis*, *M. abscessus*, *M. avium*, *M. marinum* and *M. smegmatis*.

This work represents 206 CCS values for uniquely identified mycolic acids from 5 mycobacterial species and 6 MAME families analysed in the positive ion mode and obtained on a drift tube instrument operated with nitrogen drift gas (^DT^CCS_N2_). Using the solvents required for chromatography, across all 5 mycobacterial species, ~ 76% and ~ 24% of the total ion abundance of corresponded to mycolic acid methyl ester species with proton [M + H]^+^ and sodium [M + Na]^+^ adducts, respectively (Fig. 2A). In previous literature, most MAMEs are reported as sodium or ammonium adducts^36^. In contrast, we observe that the large majority of adducts are [M + H]^+^, which can be explained by the preconditioning of the LC system by phosphoric acid; this was performed to reduce the presence of ions, such as Na^+^ and K^+^.

In terms of mycolic acids categories, carboxy; methoxy; keto; epoxy/w1-methoxy; alpha’ and alpha were annotated. More particularly, in Mtb, alpha mycolic acid methyl esters represent 55%, keto mycolic acid methyl esters represent 39% and methoxy mycolic acid methyl esters represent 6% of the total MAME ion abundance measured. In *M. abscessus*, alpha mycolic acid methyl esters represent 55%, alpha’ mycolic acid methyl esters represent 32%, epoxy/w1 methoxy mycolic acid methyl esters represent 9% and carboxy mycolic acid methyl esters represent 5% of the total MAME ion abundance measured. In *M. avium*, alpha mycolic acid methyl esters represent 44%, epoxy/w1 methoxy mycolic acid methyl esters represent 6% and carboxy mycolic acid methyl esters represent 50% of the total MAME ion abundance measured. In *M. marinum*, alpha mycolic acid methyl esters represent 40%, keto mycolic acid methyl esters represent 52%, methoxy mycolic acid methyl esters represent 9% and carboxy mycolic acid methyl esters represent 8% of the total MAME ion abundance measured. In *M. smegmatis*, alpha mycolic acid methyl esters represent 58%, alpha’ mycolic acid methyl esters represent 14%, and epoxy/w1-methoxy mycolic acid methyl esters represent 28% of the total MAME ion abundance measured. These observations are summarized in the histogram in Fig. 2B and are in agreement with the thin layer chromatography from growing mycobacteria (Figs. S1 and S2). These distributions correlate well with those found in the literature using conventional methods^14,37–41^.

### IM-MS correlation

**Figure 3:**
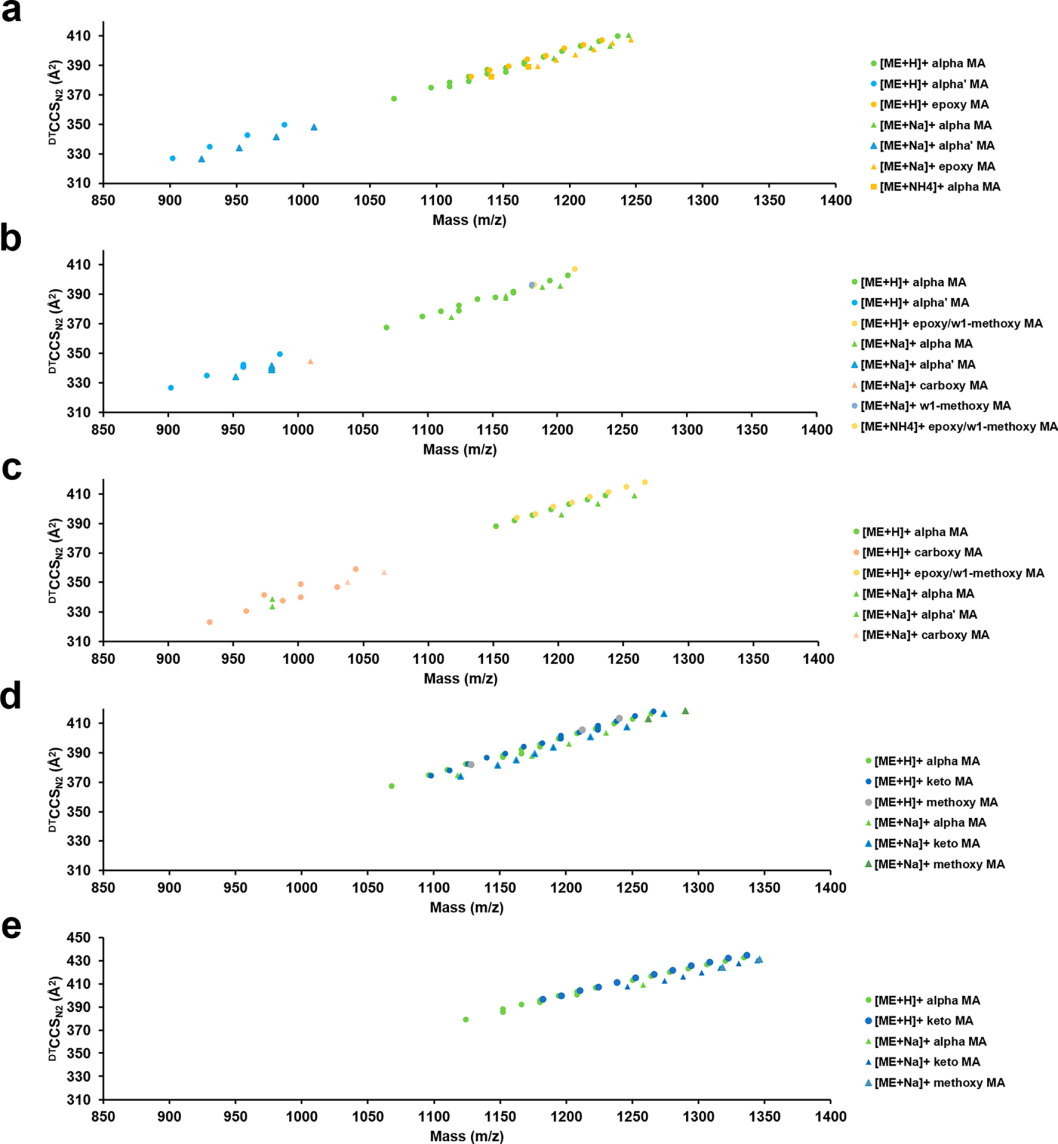
Quantitative correlations within the cation data for mycolic acid methyl esters for (**a**) *M. smegmatis*, (**b**) *M. abscessus*, (**c**) *M. avium*, (**d**) *M. marinum,* and (**e**) *M. tuberculosis*. The colours correspond to mycolic acid methyl ester modifications, whereas the shapes correspond to cation type, as specified in the corresponding panel legends.

In this work, all MAME classes exhibit a positive mobility-mass correlation in conformational space analyses, as typically observed with lipid classes in other studies. Within the MAME lipid trendline, unique families could be further differentiated by their respective ^DT^CCS_N2_ information, as each family exhibited an average CCS increase in 0.1–0.2 Å^2^ per mass unit within the investigated mass range of 850–1400 *m*/*z* (Fig. 3, Supplementary Table 1). A closer examination of the IM-MS data shows a regular increase in size for each of the mycolic acid methyl ester families with a corresponding slope, ^DT^CCS_N2_ vs. mass, ranging from 0.1–0.2 Å^2^ per mass unit.

The CCS/mass relationship presented here describes MAMEs differing only in chain length, decorations, or adduct type and can be used to identify lipid features that are ambiguous to characterize by mass alone or conformations.

For protonated adducts, the same mass *m*/*z* can correlate to more than one CCS. These findings can also be observed for the alpha MAMEs in *M. marinum* at *m*/*z* 1152.202 correlated to CCS at 388.17 Å^2^ and 386.96 Å^2^, at *m*/*z* 1166.18 correlated to CCS at 389.36 Å^2^ and 392.10 Å^2^, at *m*/*z* 1180.232 correlated to CCS at 395.7 Å^2^ and 394.27 Å^2^, and for keto MAMEs at *m*/*z* 1224.257 correlated to CCS at 406.75 Å^2^, 408.12 Å^2^, 405.77 Å^2^, 407.19 Å^2^, 408.17 Å^2^, 405.32 Å^2^. In *M. tuberculosis*, alpha MAMEs at *m*/*z* 1152.201 correlated to CCS at 385.49 Å^2^ and 388.17 Å^2^, and *m*/*z* 1180.232 correlated to CCS at 395.7 Å^2^ and 394.27 Å^2^.

**Figure 4:**
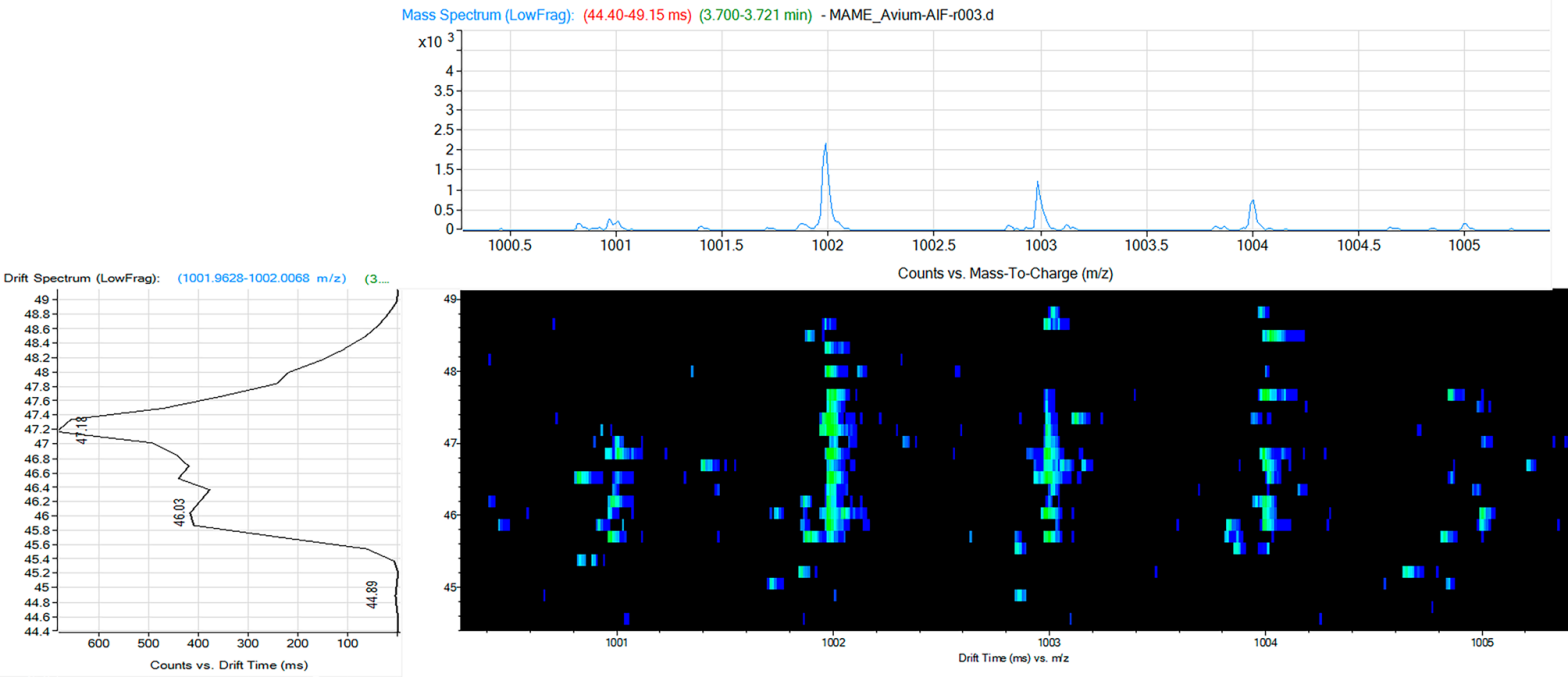
Selected example of a 2D IM-MS spectrum for carboxy mycolic acid methyl esters from *M. avium* showing separation of isobaric lipids. The top spectrum depicts the ion counts versus the mass-to-charge ratio (*m/z*), and the left spectrum depicts the drift spectrum in counts versus drift time (ms).

As in *M. avium*, these data indicate that for some carboxy MAMEs, a *m/z* can correlate to more than one CCS, such as for *m/z* 1001.987 with CCS at 340.01 Å^2^ and CCS at 348.89 Å^2^ (Fig. 4), suggesting that this lipid can adopt a different conformation in the gas phase. This information may indicate that the carboxy position is different in the meromycolic chain. A similar conclusion can be drawn for alpha’ mycolic acids, in which the double bond found in the meromycolic acid chain could be in different positions. As a result, the conformation of the meromycolic chain and therefore the CCS changes; however, the *m/z* remains the same, giving new insights into the complexity of mycolic acids.

In this study, 206 ^DT^CC_N2_ measurements are presented for the MAMEs for 5 mycobacterial species representing 6 classes of decorations. This correlation *m/z* to CCS is unique to MAMEs and provides resources for future investigations. In addition, using this approach, we observed that MAMEs can adopt multiple CCS values, which could result from different conformations in the gas phase. The majority of isomeric mycolic acids belong to the alpha, keto and carboxy classes. Compared to the acyl chain length, the degree of unsaturation is four times more impactful on CCS^26,28^; thus, some MAMEs are more likely to form isomers, resulting in the same *m/z* but different CCS values. In conclusion, this is the first study reporting on a workflow and the use of IM-MS on MAMEs from 5 different mycobacterial lipids, providing a resource for researchers.

Overall, this study demonstrates that IM-MS improves separation of mycolic acids, especially between the different isomeric forms that share the same molecular weight. Considering the important roles of mycolic acids in mycobacterial virulence and their differential distribution across mycobacterial species, accurate separation and characterisation of mycolic acids would greatly help the research in this field. As demonstrated in this study, IM-MS could be a powerful tool for this purpose based on its accuracy and specificity compared with conventional mass spectrometry or ion mobility spectrometry alone. Specifically, in LC-IM-MS studies CCS values are being increasingly leveraged as molecular identifiers that provide an orthogonal dimension of information to mass and retention time, and many software workflows such as the screening workflow shown here are being adapted to accommodate filtering on CCS values for increased specificity for both targeted compound detection and unknown compound identification. To enable the mycobacterial research community, this study presents a database of mycolic acids from various mycobacterial species and an example workflow of data analysis for such purposes. We hope to encourage future studies on the species-specific mycolic acid profiles and their functions in mycobacterial species. However, validation of CCS values is restricted to the limited number of lipid standards that are commercially available for mycolic acids, making one of the limitations for this study.

## Material and methods

### Bacterial strains and growth conditions

*Mycobacterium tuberculosis* reference strain H37Rv, *M. abscessus* subsp. *abscessus* ATCC 19977, *M. avium* subsp*. avium* Chester ATCC 25291, *M. marinum* M and *M. smegmatis* mc^2^155 were grown in Middlebrook 7H9 liquid medium supplemented with 0.2% glycerol (w/v) and 10% enrichments containing BSA (Sigma) 5% (w/v), glucose (Sigma) 2% (w/v) and NaCl (Sigma) 0.85% (w/v) and left at + 37 °C and + 30 °C for *M. marinum* M to reach OD ~ 0.8–1, representing the mid-logarithmic phase of growth. To monitor the growth of the bacteria, control flasks were inoculated at an OD_600_ of 0.01 for the *Mycobacterium tuberculosis* reference strain H37Rv and an OD_600_ of 0.001 for *M. abscessus* subsp. *abscessus* ATCC 19977, *M. avium* subsp*. avium* Chester ATCC 25291, *M. marinum* M and *M. smegmatis* mc^2^155 and grown in 7H9 liquid medium supplemented with 0.2% glycerol (w/v) and 10% enrichments containing BSA (Sigma) 5% (w/v), glucose (Sigma) 2% (w/v), NaCl (Sigma) 0.85% (w/v) and 0.05% tyloxapol (Sigma).

### Preparation of mycolic acid methyl esters

Mycolic acid methyl esters (MAMEs) were prepared as described by Bottai et al.^42^. Briefly, heat-killed mycobacteria were first washed 3 times with PBS. The pellets were then delipidated by CHCl_3_/MeOH 1:2 (v/v) extraction for 12 h at room temperature followed by one CHCl_3_/MeOH 1:1 (v/v) extraction and one CHCl_3_/MeOH 2:1 (v/v) extraction for 3 h at room temperature. To analyse the cell wall mycolic acids, the delipidated bacterial pellets were mixed with 2 ml 15% tetrabutyl ammonium hydroxide (TBAH) solution and incubated at 100 °C for 5 h. After cooling to room temperature for 1 h, the samples were incubated with 2 ml of CHCl_3_ and 100 μl of iodomethane for 1 h for methylation of the released mycolic acids. The organic lower layer after centrifugation at 2000×*g* at 4 °C for 10 min was collected and dried under a stream of N_2_. Dried methylated mycolic acids were resuspended in 3 ml diethyl ether and mixed thoroughly. All supernatants were collected after centrifugation at 2000×*g* at 4 °C for 10 min, transferred into a new glass tube and dried under a stream of nitrogen. Pellets containing MAMEs were resuspended in 50 μl of CHCl_3_ and 5 ml of MeOH and left at 4 °C overnight for precipitation. MAMEs were collected after centrifugation at 2000×*g* at 4 °C for 10 min and dried under a stream of nitrogen. The purified MAMEs were suspended in CHCl_3_ at a final concentration of 10 mg/ml and analysed by IM-MS in the positive ion mode.

### Instrumentation

The instrumentation and experimental procedures are based on the work from Leaptrot et al.^26^. Briefly, a low-field drift-tube-based 6560 Ion Mobility LC/Q-TOF mass spectrometer (Agilent Technologies) was utilized to acquire accurate mass and CCS measurements from MAME samples. The instrument consists of an orthogonal electrospray ionization (ESI) source (Agilent Jet Stream) with a heated sheath gas nebulizer for desolvation and focusing of ions at atmospheric pressure. A single bore, resistively coated, glass capillary was used to transfer ions into the vacuum system. As ions exit the transfer capillary, they are directed by a high-pressure ion funnel into a trapping funnel with two wire grids for ion trapping and gating. Ions accumulate in the trapping funnel and are subsequently pulsed into the uniform field drift tube, which is approximately 78 cm in length. An additional rear ion funnel refocuses ions as they exit the drift tube and pass to a lower pressure region via a hexapole ion guide. Ions passed through a quadrupole mass filter and collision cell before mass measurement was performed in an orthogonal time-of-flight (TOF) mass spectrometer.

### Experimental parameters

Data were acquired across all samples using UHPLC-IM-QToF. Chromatographic separation was performed using an Agilent 1290 Infinity II system (Agilent Technologies). Reversed-phase chromatography was performed using an InfinityLab RRHD Eclipse Plus C18 column, 2.1 × 50 mm, 1.8 µm (Agilent Technologies). The LC system was first preconditioned with phosphoric acid before sample acquisition. For this procedure, the LC was disconnected from the mass spectrometer, and the LC system and column were washed overnight with 0.5% (v/v) phosphoric acid at 0.1 mL/min in water/acetonitrile 20:80 (v/v), followed by a wash with LC/MS grade water for 4 h until the pH was neutral and finally reconnected to the MS system. The column temperature was set at 65 °C. Mobile phase A consisted of water/acetonitrile 40/60 + 0.1% formic acid + 10 mM ammonium formate, and mobile phase B consisted of isopropanol/acetonitrile 90/10 + 0.1% formic acid + 10 mM ammonium formate. The following gradient was applied at a flow rate of 0.4 ml/min: 0 min, 43% B; 0–0.55 min, 43% B; 0.55–3 min, 54% B; 3–3.2 min, 70% B; 3.2–4.5 min, 100% B; 4.5–10 min, 100% B; 10–10.5 min, 43% B; 10.5–12 min, 43% B. Mass axis calibration was achieved by continuous infusion after the chromatography of a reference mass solution using an isocratic pump connected to an ESI ionization source operated in positive-ion mode.

For all experiments, source conditions remained constant to ensure that the single field calibration could be used to measure CCS across all acquired data^43^. The following parameters were used: sheath gas temperature, 300 °C; nebulizer pressure, 35 psig; sheath gas flow, 11 l min^−1^; capillary voltage, 3500 V; nozzle voltage, 1000 V; and fragmentor voltage, 400 V. The data were collected in profile 2 GHz (extended dynamic range) mode.

### IM-QToF All Ions MS/MS data acquisition

With the chromatographic conditions described fragmentation data were acquired using an “All Ions MS/MS” data independent acquisition approach with two alternating experiments. The first experiment was used to acquire full spectrum ion mobility data of precursors using no collision energy. The second experiment was then employed to collect fragmentation information across the same mass range as the previous experiment by applying a collision energy of 42 eV. Ion mobility acquisition settings were as follows: mass range 800–1400 *m/z*, frame rate 2.5 frames per second, maximum drift time 100 ms, trap full time 20,000 ms and trap release time 150 ms.

### QToF iterative MS/MS

MS/MS was performed only on a pooled sample to acquire data-dependent fragmentation data. Fragmentation was performed in the collision cell following quadrupole isolation of precursor ions with a preference for mycolic acids using a preferred inclusion list. Alternate scans were performed first in TOF-only mode to collect and analyse full-spectrum MS precursor data and for mass axis calibration, a second scan with a collision energy of 20 eV and a third scan with an increased collision energy of 42 eV. Data acquisition speed was optimized for collision-induced dissociation (CID) experiments based on chromatographic peak and number of potential mycolic acid coeluting in the chromatographic space. Thus, 10 spectra/second was used to ensure sufficient MS/MS scans were acquired. In this mode, the pooled sample was injected multiple times, thereby significantly improving the coverage of precursors being selected for fragmentation.

## Supplementary Information

Supplementary Figures.

Supplementary Table 1.

## Data Availability

The datasets used and analysed during the current study are available from the corresponding author on reasonable request.
