# Supplementary material for: Ion mobility mass spectrometry for the study of mycobacterial mycolic acids
Source: Sci Rep. 2023 Jun 27;13:10390. doi: 10.1038/s41598-023-37641-9 (PMC10300000; doi:10.1038/s41598-023-37641-9)
Supplement: Supplementary file 1 — Supplementary Figures. [file 41598_2023_37641_MOESM1_ESM.docx]

**Ion mobility mass spectrometry for the study of mycobacterial mycolic acids**

Yi LIU^1^, Nadhira KAFFAH^1^, Sufyan PANDOR^2^, Mark J. SARTAIN^3^, and Gerald LARROUY-MAUMUS^1†^

^1^Centre for Bacterial Resistance Biology, Department of Life Sciences, Faculty of Natural Sciences, Imperial College London, London, SW7 2AZ, UK

^2^ Agilent Technologies, Inc., Cheadle, UK

^3^ Agilent Technologies, Inc., Santa Clara, CA, 95051


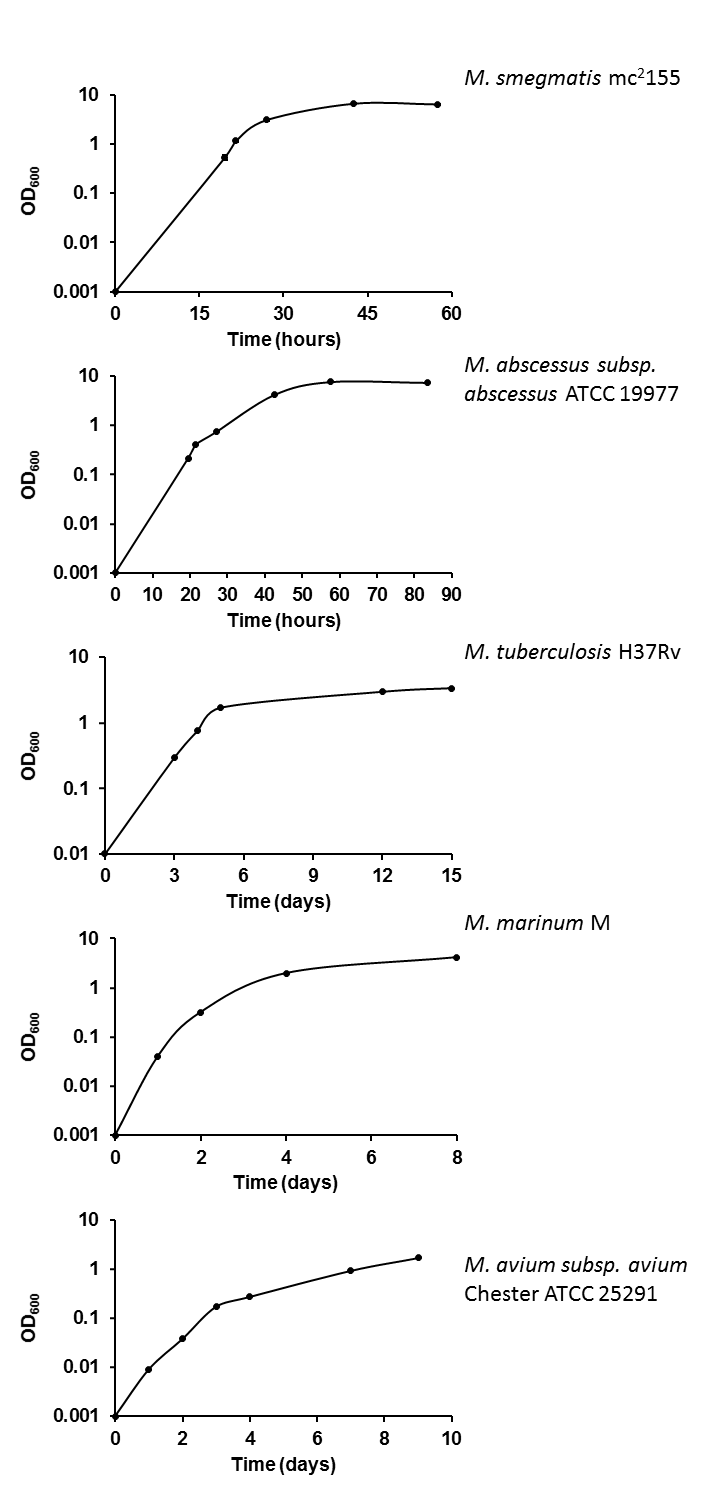


**Figure S1: Growth of mycobacteria used in this study.** Data are the means ± SD of duplicates and are representative of three independent experiments.


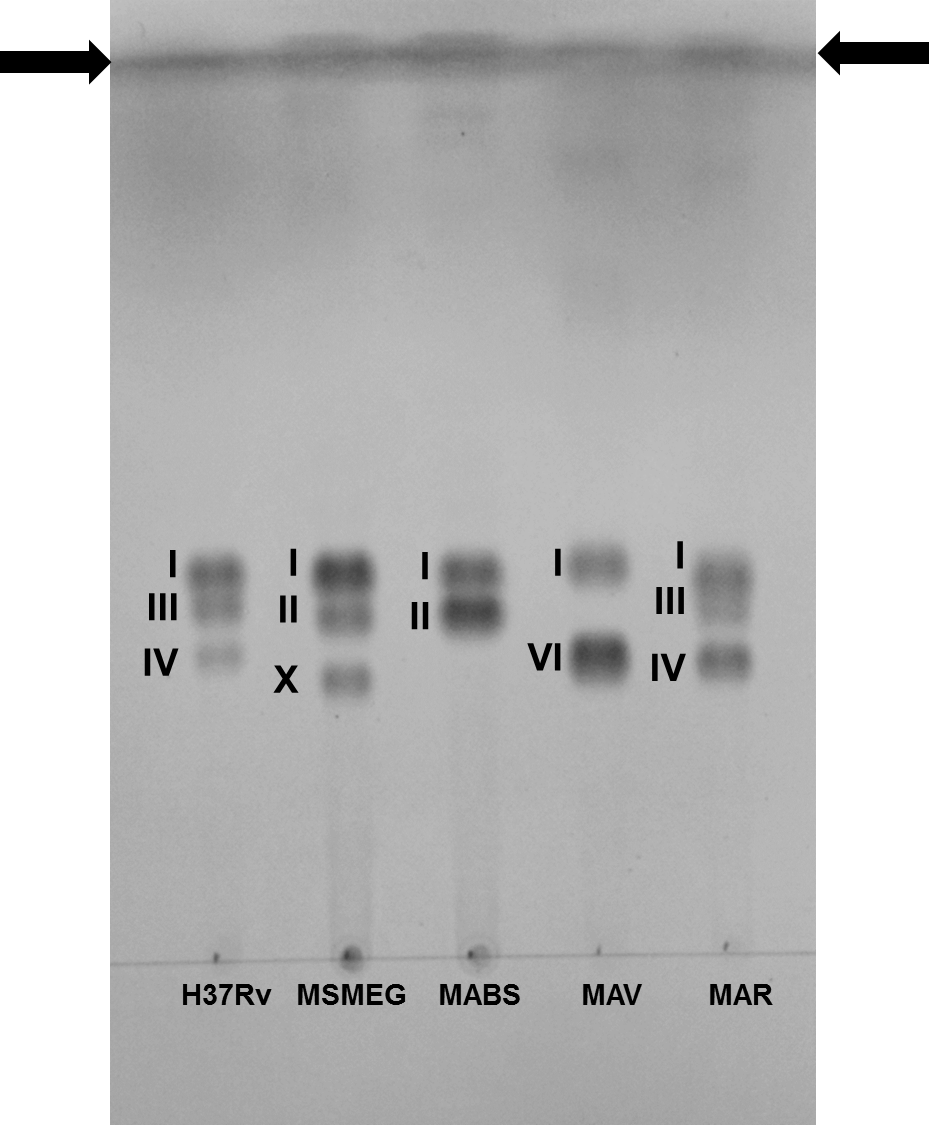


**Figure S2: Thin layer chromatography of the mycolic acid methyl esters from the five mycobacterial species presented in this study**: I, α-mycolates; II, α’-mycolates; III, methoxymycolates; IV, ketomycolates; VI, carboxymycolates; X, epoxymycolates/w-1-methoxymycolates. 20 μg of MAMEs suspended in chloroform at a concentration of 10 mg/mL were loaded into the thin layer chromatography silica gel 60; resolved using the solvent system petroleum ether/diethyl ether (90/10,v/v), visualized by charring molybdophosphoric acid 5% in ethanol, followed by heating. The arrow indicates the solvent front. *Mycobacterium tuberculosis* H37Rv (H37Rv); *Mycobacterium smegmatis* mc^2^155 (MSMEG); *M. abscessus* subsp. *abscessus* ATCC 19977 (MABS); *M. avium* subsp. *avium* Chester ATCC 25291(MAV); *M. marinum* M. Annotations are based on relative retention factor and Laval et al., ^1^.

**A**

**
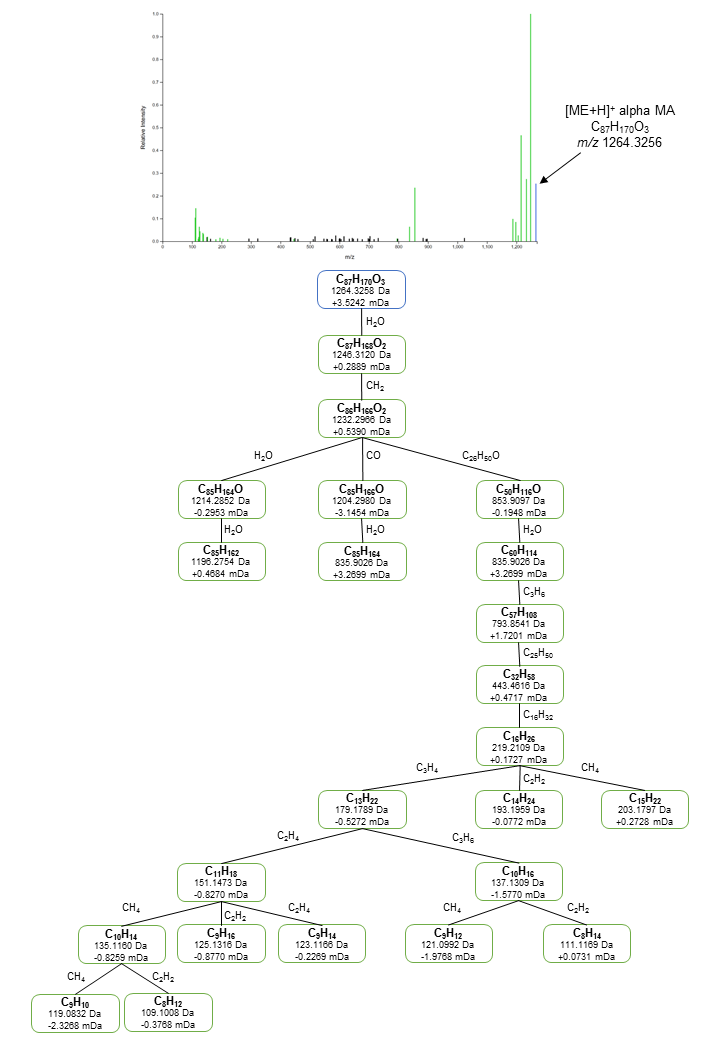
**

**B**

**
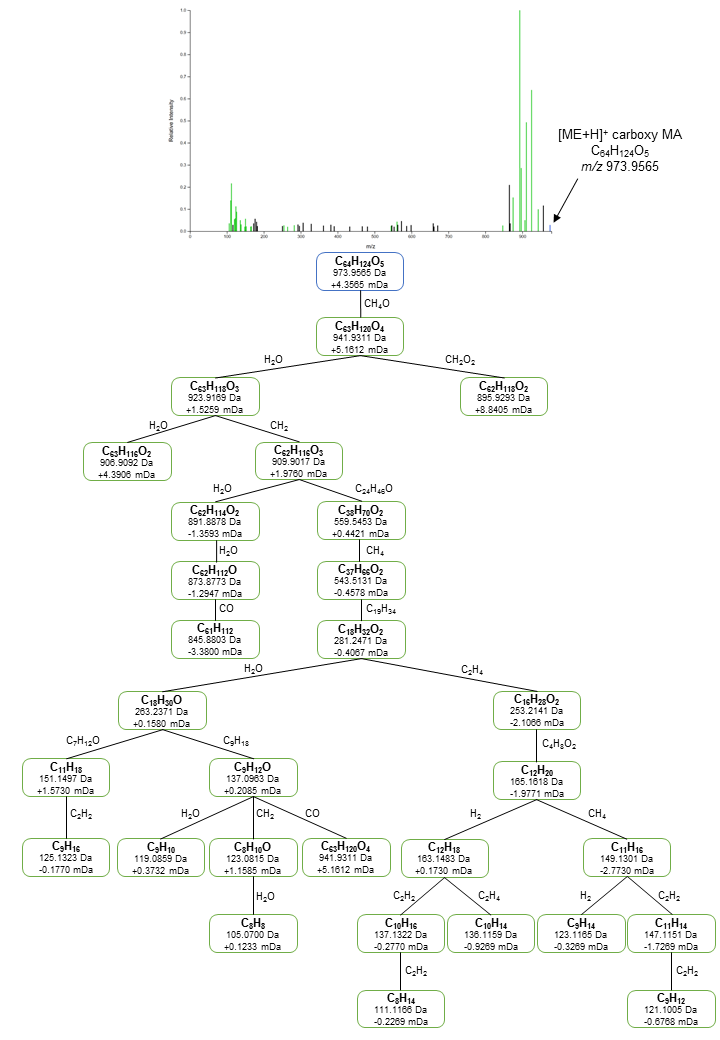
**

**C**

**
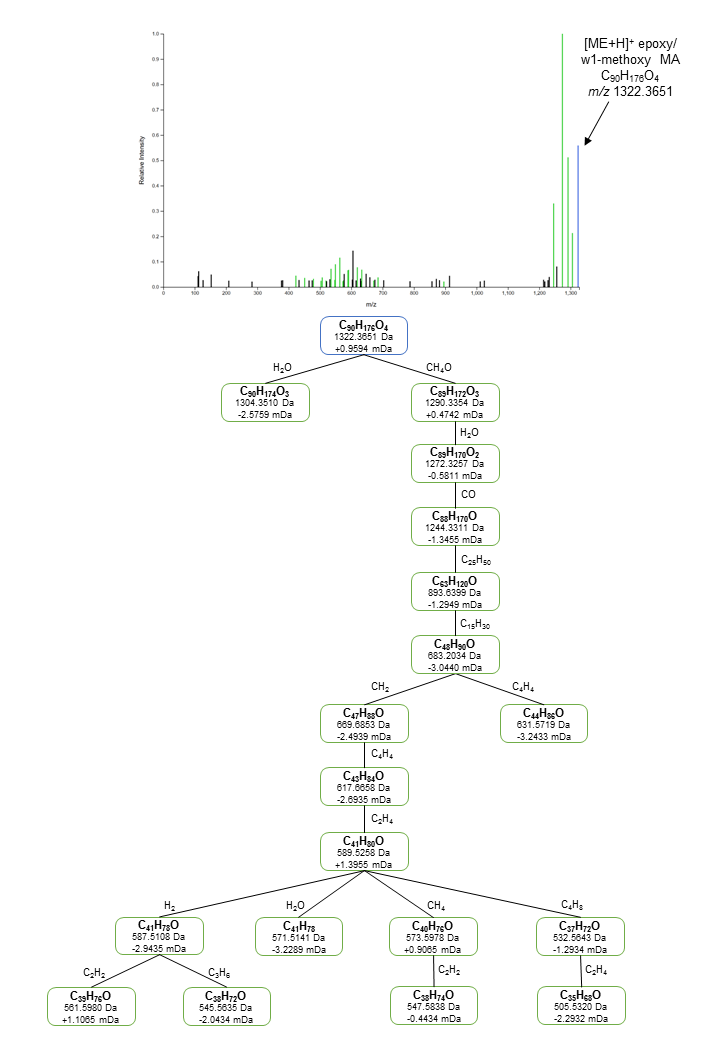
**

**D**

**
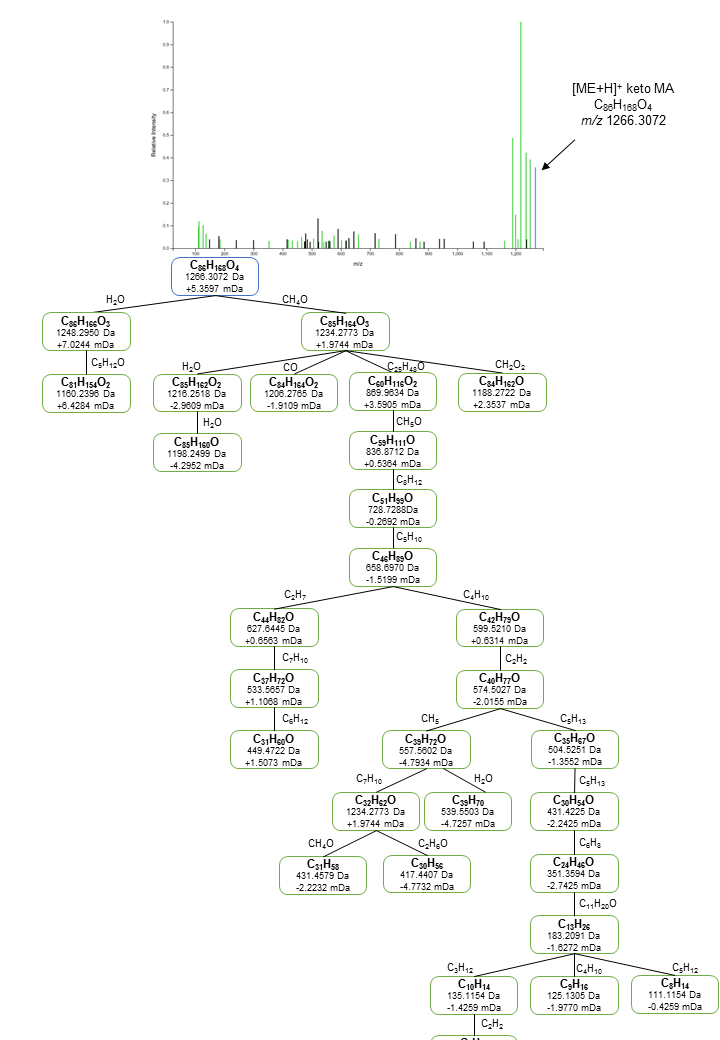
**

**E**

**
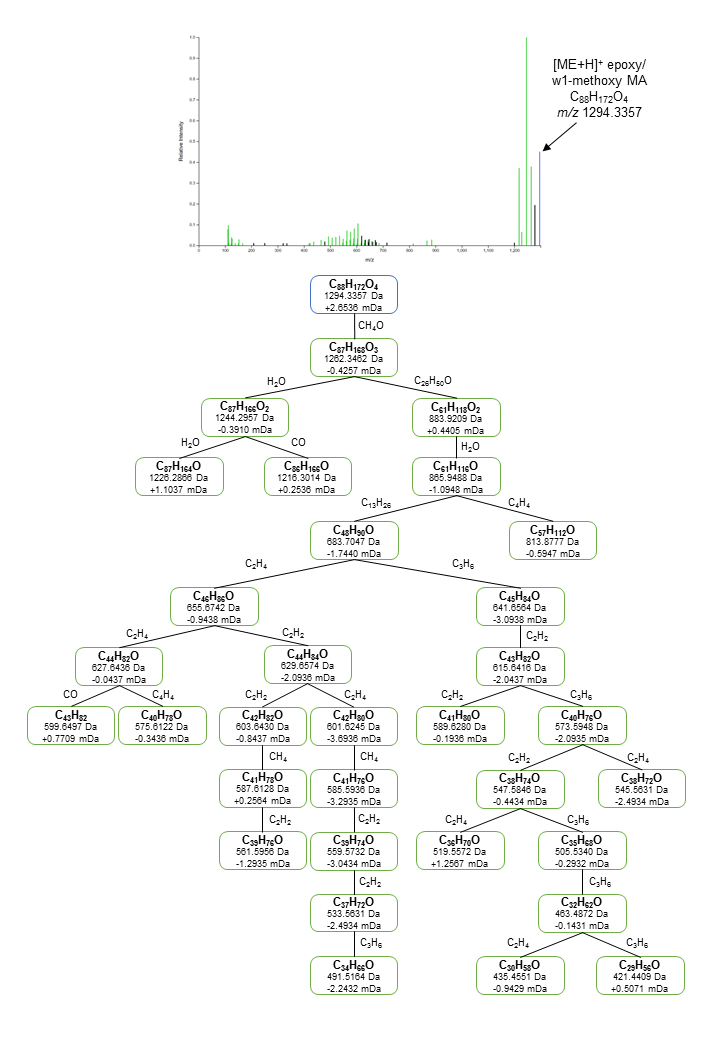
**

**Figure S3: : Example of MS/MS mass spectra in the positive ion mode.** Peaks at *m*/*z* 1264.3256 assigned to [ME+H]+ alpha MA C_87_H_170_O_3_ (**A**), *m*/z 973.9585 assigned to [ME+H]+ carboxy MA C_64_H_124_O_5_ (**B**), *m*/*z* 1322.3651 assigned to [ME+H]+ epoxy/w1-methoxy MA C_90_H_176_O_4_ (**C**), *m*/*z* 1266.3072 assigned to [ME+H]+ keto MA C_86_H_168_O_4_, (**D**) and *m*/*z* 1294.3357 assigned to [ME+H]+ epoxy/w1-methoxy MA C_88_H_172_O_4_ (**E**). In the full spectrum MS/MS, the green peaks represent fragments that come from the precursor ion which is displayed in the blue colour. The fragmentation tree showing a proposed fragmentation pattern corresponding to the green peaks observed in the MS/MS spectra.

**Reference**

1 Laval, F., Laneelle, M. A., Deon, C., Monsarrat, B. & Daffe, M. Accurate molecular mass determination of mycolic acids by MALDI-TOF mass spectrometry. *Anal Chem* **73**, 4537-4544, doi:10.1021/ac0105181 (2001).
